# Visual outcomes of dense pediatric cataract surgery in eastern China

**DOI:** 10.1371/journal.pone.0180166

**Published:** 2017-07-03

**Authors:** Fangqin Ma, Meiyu Ren, Lihua Wang, Qi Wang, Jingli Guo

**Affiliations:** Department of Ophthalmology, Shandong Provincial Hospital Affiliated to Shandong University, Jinan, P. R. China; Bascom Palmer Eye Institute, UNITED STATES

## Abstract

**Purpose:**

To evaluate the visual outcomes of dense pediatric cataract surgery in eastern China.

**Methods:**

Medical records of children who underwent surgery for dense unilateral or bilateral pediatric cataract in Shandong Provincial Hospital between January 2007 and December 2012 were collected. Patients who cooperated with optical correction and aggressive patching of the sound eye and who had a minimum postoperative follow-up of more than 2 years were included. Risk factors for poor visual outcomes were analyzed.

**Results:**

Of the 105 eligible patients (181 eyes), 76 had bilateral cataract, and 29 unilateral. With a mean follow up of 46.77 mo (range 24.0~96.0 mo), the final best corrected visual acuity (BCVA) of 158 eyes were recorded, and 4.43% (7/158) achieved 0.1 logarithm of the minimum angle of resolution (logMAR) or better; 15.19% (24/158) obtained a BCVA between 0.1 logMAR and 0.3 logMAR; 18.99%, (30/158) between 0.3 logMAR and 0.5 logMAR; 46.84% (74/158), between 0.5 logMAR and 1 logMAR; 14.55%, worse than 1 logMAR. The mean BCVA of the patients who underwent lensectomy before 3 months of age was significantly better than that of patients who underwent lensectomy between 3 and 12 months (p = 0.001). In the same lensectomy age groups, the final BCVA of the children in the bilateral and unilateral groups did not differ significantly (P>0.05). Lensectomy after 3 months of age, postoperative complications, strabismus and nystagmus were shown to be risk factors for poor visual outcomes.

**Conclusions:**

Lensectomy before 3 months of age, IOL implantation, proper managing of postoperative complications, early optical correction and aggressive postoperative patching of the sound eye would increase the final BCVA for patients with dense pediatric cataract.

## Introduction

Pediatric cataract accounts for 22% of visual impairment in children in the African region, 21.3% in the Western-Pacific region, 13.6% in the South-east Asian region, and 15.2% in the European region. As a treatable cause of visual handicap in childhood, pediatric cataract is a priority of the “VISION 2020 Global Initiative”[1]. The aim of pediatric cataract surgery is to provide and maintain a clear visual axis and a focused retinal image [2]. Proper management of pediatric cataract is crucial to the visual development and rehabilitation of affected children. A better understanding of the disease and advances in surgical techniques has greatly increased the success rate of pediatric cataract surgeries. However, in some developing countries, because of relatively late detection and diagnosis, poor medical conditions and poor compliance with long-term follow-up, the visual prognosis for patients with pediatric cataract is relatively poor compared with that of patients in developed countries. Some studies [3, 4] have focused on the current situation of pediatric cataract treatment in some parts of China, but few studies with large samples have been reported. The purpose of this study was to evaluate the visual outcomes of dense pediatric cataract surgery between January 2007 and December 2012 at Shandong Provincial Hospital affiliated to Shandong University, a large central medical center in eastern China.

## Methods

### Patients

This study was approved by the institutional review board of Shandong Provincial Hospital affiliated to Shandong University, China, and conformed to the guidelines of the Declaration of Helsinki. We prospectively collected the data of all the children who were diagnosed with dense pediatric (congenital or developmental) cataract and had surgery between January 2007 and December 2012 at the hospital. Routine treatment was performed for all subjects, and no interventions were included in this work. The inclusion criteria were as follows: (1) total cataract or axial opacities of the lens that were large enough to obscure the visual axis; (2) extracapsular lensectomy with/without anterior vitrectomy and with/without intraocular lens (IOL) implantation; (3) early postoperative optical correction for the aphakic eyes; (4) aggressive postoperative patching of the sound eye; (5) minimum postoperative follow-up of no less than 2 years. Those patients with severe general malformations such as anterior segment dysgenesis, congenital glaucoma, persistent hyperplasia of primary vitreous (PHPV), congenital microphthalmos, previous ocular trauma, retinal detachments or other anomalies that would influence the visual development or visual assessment, poor compliance with subsequent therapy such as refractive correction and patch therapy were excluded. The authors had access to information that could identify individual participants during or after data collection.

### Groups and preoperative examinations

Prior to surgery, all patients were examined and diagnosed by the same ophthalmologist (L.W.). Routine preoperative examinations included assessment of BCVA if possible, intraocular pressure (IOP) measurement, slit-lamp inspection, fundus examination and an A/B-ultrasonography scan. Young children who were uncooperative were examined under sedation with 10% chloral hydrate. The density and extent of the opacity in the visual axis were assessed by slit-lamp (desk-mounted or handheld) and the red reflex test with a direct ophthalmoscope. Healthy children with dense cataracts underwent surgery as early as 6 weeks of age.

### Surgery

Surgery was performed under general anesthesia as soon as the dense cataract was diagnosed. In preparation for surgery, eye drops of levofloxacin (0.5%, 5 ml, Santen Pharmaceutical, Japan) were applied 4 times a day for at least 3 days to keep the conjunctival sac relatively sterile. Simultaneous surgery was performed for bilateral cases. Extracapsular lensectomy with primary posterior capsulorhexis and anterior vitrectomy via limbal approach was performed. A limbal incision of 2.4 mm was made, high-molecular-weight viscoelastic material was injected into the anterior chamber, and anterior circular capsulorhexis was carried out. Phaco-aspiration was used to perform the extracapsular lensectomy. Then, posterior capsulorhexis of approximately 4 mm in diameter was made, and central anterior vitrectomy was preferred for patients younger than 6 years of age. The posterior capsule was left intact in children above 6 years. Primary IOL implantation was performed in children over 2 years of age with bilateral cataract and in those over 1 year of age with unilateral cataract. In most patients who were left aphakic, a secondary IOL implantation was performed over 2 years of age if the status of the aphakic eye was suitable for surgery (normal IOP, perfect dilated pupil size and the residual lens capsule could provide a stable support for the IOL). The IOL power was calculated based on the SRK-II formula targeting 3–4 diopter (D) undercorrection for children aged 1–2 years and 2–3 D undercorrection for children aged above 2 years [3–5]. When the autokeratometry could not be applied, an average power of 43 D was chosen for theoretical keratometry readings. IOL (AcrySof IQ SN60WF, AcrySof MA60AC, Alcon Laboratories, Inc., USA) was implanted in the capsule or ciliary sulcus for either primary or secondary implantation according to the status of capsule. After swapping out viscoelastic material, the limbal incision was sutured with 10–0 nylon suture.

### Postoperative management

After the surgery, topical antibiotics, corticosteroids, and cycloplegics were applied for several weeks. Topical steroids were used more aggressively in children who were younger, had a more serious intracameral inflammatory reaction, or had undergone IOL implantation. Sometimes, for very young children, intraoperative steroids were given by the anesthesiologist to prevent possible laryngeal edema or spasms of the respiratory tract after tracheal intubation, and postoperative steroids may also have been given to the children who had very serious intracameral inflammatory reaction. Postoperative follow-ups were scheduled for 1 day, 1 week, 2 weeks, 1 month, 3 months, and every 3 months after surgery for 1 year and twice every year thereafter.

For aphakic children, spectacles with press-on optics were prescribed within 1 week after surgery. Residual refractive errors in children with IOLs were corrected with spectacles. For very young children, overcorrection with a positive lens was applied to provide good near focus. Cycloplegic refraction was performed every half year for all the children. Patching of the sound eye was indicated in cases of unilateral cataract or asymmetric bilateral cataract. The patching time daily was determined by the degree of amblyopia and the age of the child.

At all visits, routine examinations included BCVA, slit-lamp or operating microscope examinations, fundus examinations, intraocular pressure, and examinations for strabismus and nystagmus. IOP was measured by Schiotz tonometry under sedation in the eye for young children and pneumatic tonometer for children who would cooperate. Chloral hydrate (CH) combined with topical Alcaine (0.5% proxymetacaine hydrochloride, Alcon, Inc., Belgium) was usually used in our clinic for the routine eye examinations of young children. A dosage of 50 mg/kg is safe, with little risk of respiratory depression or other harm, and provides sufficient sedation for slit-lamp, fundus and intraocular pressure examinations, and even ultrasound tests. General anesthesia was used if more tests were needed. To improve safety, these guidelines were followed: 1. medication history was asked for to exclude those children who may have been difficult to sedate with CH; 2. patients had continuous cardiorespiratory monitoring with pulse oximetry; 3. the maximum total dose of CH was not to exceed 100 mg/kg or 2 g; 4. discharge occurred when the patient returned to baseline or to a sleepy but easily arousable state. To improve the success rate, these guidelines were followed: 1. no other fluids were given 2–3 hours before CH; 2. CH mixed with 25% glucose was administered around the usual nap time of the patients; 3. after CH was administered, a waiting time of 30 minutes was required before any examination. In our hospital and many hospitals in China, CH instead of phenobarbital, dexmedetomidine, diazepam or any other benzodiazepines was used for sedation in children. This may be due to a prescribing habit, but the easy and non-invasive mode of administration of CH also plays a role.

The method of evaluating visual acuity varied, according to the age of children and the level of cooperation. If optotype methods such as Snellen’s chart, Teller acuity, and Allen pictures failed, fixation patterns such as Square Wave Grating Paddles (Cat No 2533) or sweep-VEP for the younger children were used.

Postoperative complications such as fibrin formation, pupillary synechia, pupil decentered causing occlusion of the visual axis in the natural pupil, IOL displacement, secondary glaucoma, and visual axis opacification (VAO) were treated in time by drugs or surgery if necessary.

### Data collection and statistical analysis

The following data were collected: age at the time of surgery, serious postoperative complications, development of strabismus and nystagmus, periods of follow-up, and the final BCVA. BCVA and its related risk factors were statistically analyzed after conversion to logMAR. SPSS version 18.0 was used to perform the statistical analysis. The t-test, rank sum test, variance analysis and ordinal regression analysis were used to analyze the data.

## Results

### Patient characteristics

A total of 105 children (181 eyes) were included in the study: 66 boys (62.86%) and 39 girls (37.14%). Patient data are given in Table 1. There were 76 children (152 eyes) in the bilateral cataract group and 29 children (29 eyes) in the unilateral group. The mean age at lensectomy was 20.3 mo (range 1.3~96.0 mo). The mean follow-up period for all children was 46.8 mo (range 24.0~96.0 mo). At the final follow-up, 83 (79.1%) children had an IOL implanted.

**Table 1:** Patient characteristics.

Category	Bilateral	Unilateral	Total	P-value
No. of patients	76 (72.4%)	29 (27.6%)	105	
No. of eyes	152 (84.0%)	29 (16.0%)	181	
Male/female	52/24	14/15	66/39	
Mean age at time of lensectomy (mo)	17.8 (1.3~96.0)	26.9 (1.5~72.0)	20.3(1.3~96.0)	0.013
No. of patients with IOL implantation at last follow-up	59/76 (77.6%)	24/29 (82.8%)	83/105 (79.1%)	0.564
Mean follow-up period (mo)	52.76 (24~96)	31.05 (24~72)	46.77 (24~96)	

### Visual outcomes

In the last follow up, the BCVA of 90 patients (68 bilateral and 22 unilateral) was recorded as the others were not compliant with the visual assessment. The BCVA data is given in Table 2.

**Table 2:** BCVA at final follow-up.

Category	BCVA < 1 logMAR	1 logMAR ≤ BCVA < 0.5 logMAR	0.5 logMAR ≤ BCVA < 0.3 logMAR	0.3 logMAR ≤ BCVA < 0.1 logMAR	BCVA ≥ 0.1 logMAR
No. of eyes	23 (14.55%)	74 (46.84%)	30 (18.99%)	24 (15.19%)	7 (4.43%)
Bilateral	21 (15.44%)	64 (47.06%)	27 (19.85%)	18 (13.24%)	6 (4.41%)
Unilateral	2 (9.09%)	10 (45.46%)	3 (13.64%)	6 (27.27%)	1 (4.54%)

### BCVA and lensectomy age

According to the time of surgery, all patients were classified into 4 groups. The BCVA of patients in different groups are shown in Table 3. The mean BCVA of the patients who underwent lensectomy before 3 months after birth was significantly better than those who underwent lensectomy between 3 and 12 months (logMAR, 0.66±0.27 vs. 0.85±0.24, p = 0.001) in both the unilateral (p = 0.004) and bilateral (p = 0.003) groups. In the bilateral group, the mean BCVA of the patients who underwent surgery at an age above 36 mo was significantly better than that of patients who underwent surgery from 12 to 36 mo (P<0.001), while in the unilateral group, the difference was not significant (p = 0.647). For those in the same surgery age group, the final BCVA of the children in the bilateral and unilateral groups did not differ significantly (P>0.05).

**Table 3:** BCVA and lensectomy age.

Groups (lensectomy age)	Group A (<3 mo)	Group B (3~12 mo)	Group C (12~36 mo)	Group D (≥36 mo)	P-value
No. of eyes (#)	31/35	65/78	26/32	36/36	
BCVA (logMAR)	0.66±0.27	0.85±0.24	0.74±0.34	0.39±0.39	P1 = 0.001 P2 = 0.224 P3<0.001
Bilateral Group (#)	28/32	60/70	20/22	28/28	
BCVA (logMAR)	0.65±0.27	0.84±0.25	0.77±0.34	0.37±0.37	P1 = 0.003 P2 = 0.388 P3<0.001
Unilateral Group (#)	3/3	5/8	6/10	8/8	
BCVA (logMAR)	0.7±0.35	0.96±0.05	0.67±0.34	0.44±0.43	P1 = 0.004 P2 = 0.177 P3 = 0.647
P*	0.762	0.241	0.457	0.588	

^(#)^: (No. of eyes compliant with VA assessment / total number of eyes qualified);

P1: Significant difference between groups A and B;

P2: Significant difference between groups B and C;

P3: Significant difference between groups C and D;

P*: Significant differences of the mean BCVA of four age-matched groups between the bilateral group and unilateral group.

### BCVA and IOL implantation

In the 105 children (181 eyes) included in this study, 83 children (79.1%) had an IOL implanted at the last follow-up (Table 1), with 59 (77.6%) from the bilateral group and 24 (82.8%) from the unilateral group. The BCVA of patients with primary/secondary/no IOL implantation are given in Table 4. Better visual outcomes were obtained in patients with primary IOL implantation than in those with secondary IOL implantation (logMAR, 0.45±0.38 vs. 0.77±0.26, P<0.01) or those with aphakic patients (logMAR, 0.45±0.38 vs. 0.92±0.29, P<0.01). The mean BCVA was better in patients with secondary IOL implantation than in aphakic patients (logMAR, 0.77±0.26 vs. 0.92±0.29, P = 0.015). However, in both the primary and secondary IOL implantation groups, the final BCVA of patients with bilateral cataract and that of patients with unilateral cataract did not differ significantly.

**Table 4:** BCVA and IOL implantation.

Category	Bilateral group	Unilateral group	Total	P-value
Total				
No. of eyes (#)	136/152	22/29	158/181	
Mean BCVA (logMAR)	0.7±0.35 (0~1.4)	0.66±0.38 (0~1.22)	0.69±0.35 (0~1.4)	0.755
Primary IOL implantation				
No. of eyes (#)	36/38	14/19	50/57	
Mean BCVA (logMAR)	0.42±0.38 (0~1.4)	0.54±0.4 (0~1.22)	0.45±0.38 (0~1.4)	0.349
Secondary IOL implantation				
No. of eyes (#)	76/80	5/5	81/85	
Mean BCVA (logMAR)	0.75±0.27 (0.3~1.4)	0.88±0.22 (0.5~1)	0.77±0.26 (0.3~1.4)	0.26
Aphakia				
No. of eyes (#)	24/34	3/5	27/39	
Mean BCVA (LogMAR)	0.93±0.29 (0.5~1.4)	0.83±0.31 (0.5~1.1)	0.92±0.29 (0.5~1.4)	0.641
P	P1<0.01; P2<0.01; P3 = 0.015			

^#^: Number of eyes compliant with VA assessment/ total number of eyes;

P1: Significant difference between the mean BCVA of patients with primary and secondary IOL implantation.

P2: Significant difference between the mean BCVA of patients with primary IOL implantation and that of aphakic patients.

P3: Significant difference between the mean BCVA of patients with secondary IOL implantation and that of aphakic patients.

### Serious long-term complications

The incidences of serious long-term complications observed in this study are shown in Table 5. Of all 181 eyes (105 patients) operated on, 34 eyes (18.78%) presented VAO. No statistically significant difference in VAO was observed among the three different groups (p = 0.174). Three eyes of 3 patients underwent YAG-laser capsulotomy, while others were treated with membranectomy. Secondary angle-closure glaucoma occurred in 2 aphakic eyes. One patient received a bilateral lensectomy at the age of 120 days, and it was found that the IOP was elevated in the right eye because of membrane formation in the pupil 6 months postoperatively. Long-term control of IOP was achieved by membranectomy followed by application of topical steroids and cycloplegics. The other patient received bilateral lensectomy at the age of 113 days and was diagnosed with glaucoma of the right eye 11 months postoperatively. After undergoing ineffective treatment with topical anti-glaucoma drugs, he received drainage implant surgery and recovered control of his IOP.

**Table 5:** Incidence of serious long-term complications.

	Primary IOL implantation	Secondary IOL implantation	Aphakia	Total	P-value
Early-onset Glaucoma	0 (0.0%)	0	2/39 (5.13%)	2/181 (1.11%)	
VAO	11/57 (19.30%)	12/85 (14.12%)	11/39 (28.20%)	34/181 (18.78%)	0.174
IOL decentration	0	6/85 (7.06%)	0	6/181 (3.31%)	
Pupil decentration	2/57 (3.51%)	5/85 (5.88%)	2/39 (5.13%)	9/181 (4.97%)	0.807

Strabismus developed in 23.8% (25/105) of patients, with 15 esotropic cases and 10 exotropic cases. The number of patients in the unilateral group (37.9%) was significantly higher than that in the bilateral group (18.4%) (p = 0.042). In the bilateral group, 28.9% (22/76) of patients developed nystagmus. None of the patients developed nystagmus in the unilateral group.

### Risk factors for visual rehabilitation

In this study, risk factors for poor visual outcomes were analyzed (Table 6). Subjects from the bilateral congenital cataract group who had undergone lensectomy before 36 months of age were included for analysis. Of the 181 eyes, 108 eyes were included. The underlying factors associated with poor visual outcomes are shown in Table 6. Long-term complications (decentered pupil, IOL displacement, secondary glaucoma, and VAO), strabismus and nystagmus were shown to be correlated with the incidence of poor visual outcomes (all P<0.05). This study also showed that those patients who received lensectomy before 3 months of age were more likely to achieve better visual outcomes (P<0.05), while those who had surgeries between 3 months and 12 months of age or between 12 months and 36 months were not (p = 0.08).

**Table 6:** Factors associated with BCVA.

Factors	Odds Ratio	95% CI	P-value
Age at lensectomy			
<3 mo	0.055	0.011–0.270	0.000
3~12 mo	0.327	0.093–1.145	0.08
12~36 mo			
Long-term complications			
None	0.011	0.002–0.077	0.000
Mild or moderate	0.058	0.007–0.471	0.008
Serious			
Strabismus and Nystagmus			
None	0.38	0.150–0.684	0.041
Present			

CI: Confidence interval.

## Discussion

If not treated promptly, dense pediatric cataract, especially in congenital or infantile cases, may lead to profound and irreversible vision loss. Enhanced understanding of the critical periods of visual development has led to surgical interventions being deemed necessary within the first 3 months of life, possibly as early as the first 6 weeks for the proper development of binocular vision [5]. Magli [6] reported that with a mean surgery time of approximately 6 months of age, only 12% of the eyes with total cataract achieved 20/40 VA or better, while in eyes with partial cataract, the percentage was 93%. After a large cohort study with a mean follow-up of 10.6 years, Birch [7] reported that for patients with dense congenital unilateral cataract, with a mean age at surgery of 10 weeks, a mean BCVA of 0.4 logMAR was achieved. In this study, only dense pediatric cataract was included, with a mean cataract surgery age of 20.3 months. A mean BCVA of 0.69 logMAR was achieved, with 19.6% of the eyes better than 0.3 logMAR (20/40) and 38.6% of the eyes better than 0.5 logMAR (6/18).

Birch [7] reported that for patients with dense congenital unilateral cataract, there is a linear relationship between delay in surgery and long-term visual acuity outcome during the first 14 weeks of life, with an average loss of one line of visual acuity for every 3 weeks of delay. Between 14 and 31 weeks of age, there is little additional adverse effect in further delay of surgery. Similarly, in this study, the patients who underwent lensectomy before 3 months of age gained better BCVA than those who underwent lensectomy between 3 months and 12 months of age, but those who underwent lensectomy from 3 months to 12 months of age had no significant difference from those who did from 12 months to 36 months of age. However, in the bilateral cataract group, the eyes that received surgery after 36 months of age achieved better mean BCVA than those who underwent lensectomy between 12 months and 36 months of age. To explain these results, we assume it is possible that some dense pediatric cataract cases may be developmental, in which the visual function had developed to a certain extent before the dense cataract formed. Early detection followed by surgery before the end of the third month is important to decrease the risk of marked acuity loss [8]. Compared with previous studies from developed countries [7, 8, 9], delay of surgery in this study was still very common because most of our patients came from rural areas where there was a poor primary eye care system. Therefore, there is an urgent need in China for an improved neonatal screening system.

Allen reported that more than two-thirds of children with congenital cataract did not develop visual acuity better than 0.6 logMAR in their aphakic eye [10]. Primary IOL implantation is the preferred method of optical correction in older children after lensectomy [4, 11]. A large sample study by Solebo et al [12] including 221 children under 2 years of age with congenital or infantile cataract found that IOL implantation was independently associated with better visual outcomes in bilateral but not unilateral cases. He recommended that the use of IOL in cataract surgery in young children should be critically reassessed. In this study, primary and secondary IOL implantation was performed in children over 2 years old with bilateral cataract and in children over 1 year with unilateral cataract. Better visual outcomes were obtained in patients with primary IOL implantation than in those with secondary IOL implantation or with aphakic eyes, which was in accordance with the studies above.

Posterior Capsule Opacification (PCO) causes visual axis obscuration and disrupts visual rehabilitation [3, 13–15]. Posterior continuous curvilinear capsulorhexis (PCCC) and anterior vitrectomy were recommended to be performed in all children up to 6–7 years old to reduce the incidence of PCO [16, 17]. However, VAO could sometimes still be observed in them. It may be lens reproliferation, a pupillary membrane or a tissue proliferation resulting from an intracameral inflammatory reaction. In the present study, the incidence of VAO was 34 of 181 eyes (18.8%) in our study, which corresponds with reports in the literature [18–21]. Characteristics of early-onset and delayed-onset glaucoma were described by Kang et al. [22]. Early-onset glaucoma was significantly more likely to be due to angle closure than delayed-onset glaucoma. Lambert [23] reported that 14.5% of the studied subjects developed glaucoma with a median of 4.3 months after cataract surgery. Some authors [24, 25] reported that the interval between pediatric cataract surgery and the onset of open-angle glaucoma averaged from 7.4 years to 12.2 years. In the present study, early-onset angle-closure glaucoma occurred in 2 cases of aphakic eyes, and there were no cases of delayed-onset glaucoma, which may be partially due to the relatively short period of our follow-up. Hence, these patients require lifelong surveillance after cataract surgery.

Developmental strabismus or nystagmus was a clinical indicator of impending amblyopia [3, 26–28]. Children with unilateral dense cataract tended to develop strabismus; children with bilateral dense cataract tended to develop nystagmus [4, 9, 29]. Birch et al. [9] indicated that congenital cataracts with visual deprivation after more than 1.5 months were associated with a significantly higher risk for strabismus and nystagmus. Strabismus is often the presenting sign of a child with a unilateral cataract and is also frequently present preoperatively in children with bilateral cataract. In this study, 23.8% (25/105) of patients developed strabismus, and the unilateral cataract group (11/29, 37.9%) had a higher incidence than that of the bilateral cataract group (14/76, 18.4%). Additionally, 28.9% (22/76) of patients developed nystagmus in the bilateral group. The regression analysis showed that strabismus and nystagmus could be important risk factors for poor visual outcomes, which is in accordance with previous studies mentioned above. [3, 26–28]

The main limitation of this study is that the mean follow-up period of 46.8 months is a relatively short time, as over time, the visual function of the patients may change, delayed-onset open-angle glaucoma may develop, and the incidence of strabismus may increase. Even so, it may be suggested that lensectomy before 3 months of age, primary and secondary IOL implantation, early optical correction and aggressive postoperative patching of the sound eye would increase the final BCVA for patients with dense unilateral or bilateral pediatric cataract. It is important to bear in mind that surgery is just the beginning of pediatric cataract treatment, as managing postoperative complications and detecting and correcting refractive change are needed for long-term care following surgery.

## Supporting information

S1 Table Original data of all patients. (XLSX)
